# Taxonaut: an application software for comparative display of multiple taxonomies with a use case of GBIF Species API

**DOI:** 10.3897/BDJ.4.e9787

**Published:** 2016-09-30

**Authors:** Nozomi Ytow

**Affiliations:** ‡ Faculty of Life and Environmental Sciences, University of Tsukuba, Tsukuba, Ibaraki 305-8572, Japan

**Keywords:** Multiple hierarchies, Data visualization, Tree comparison, GBIF API

## Abstract

**Background:**

The Species API of the Global Biodiversity Information Facility (GBIF) provides public access to taxonomic data aggregated from multiple data sources. Each data source follows its own classification which can be inconsistent with classifications from other sources. Even with a reference classification e.g. the GBIF Backbone taxonomy, a comprehensive method to compare classifications in the data aggregation is essential, especially for non-expert users.

**New information:**

A Java application was developed to compare multiple taxonomies graphically using classification data acquired from GBIF’s ChecklistBank via the GBIF Species API. It uses a table to display taxonomies where each column represents a taxonomy under comparison, with an aligner column to organise taxa by name. Each cell contains the name of a taxon if the classification in that column contains the name. Each column also has a cell showing the hierarchy of the taxonomy by a folder metaphor where taxa are aligned and synchronised in the aligner column. A set of those comparative tables shows taxa categorised by relationship between taxonomies. The result set is also available as tables in an Excel format file.

## Introduction

Taxonomy is the discipline that manages classification of organisms, where each classification, or taxon, is examined as scientific hypothesis. A taxon is a concept that may vary with time depending on the developing understanding of nature or geological area of interest, as a subset of the natural world. Taxa, or more precisely names of taxa, are also used as building blocks to describe nature, especially in biology and environmental sciences. Information on organisms is organised and accessed using their names. The utility of an unnamed specimen is severely limited because it lacks a mechanism to access and manage any associated data, as well as the ability to locate that particular specimen at will within a large collection.

The Global Biodiversity Information Facility (GBIF) has been gathering data on organism specimens for over a decade using a consistent data format, Darwin Core (Wieczorek et al. 2012). A Darwin Core record provided in the first released schema (Darwin Core Task Group, Biodiversity Information Standards (TDWG) 2009) contains a scientific name of the specimen, depending on its level of identification which need not be to species level but can be a higher taxon name, with, optionally, a list of higher taxon names representing the classification that the identification followed. Taxonomic hierarchies behind those data are not necessarily coherent or even consistent, because data providers may choose a classification depending on the purpose of their collection. Therefore, it is necessary to coordinate those taxonomies to use specimen data stored in GBIF in a concordant manner. It is not limited to specimen data but can apply to any data indexed by biological name. GBIF’s Backbone Taxonomy (GBIF Secretariat 2013) is an effort to provide a synthetic index covering all names in GBIF data. It requires all classifications be cast into a single view, as Catalogue of Life (Roskov et al. 2016) seeks to achieve through expert panels, but the resulting taxonomy is unlikely to satisfy every user. It is challenging to provide a coherent, single taxonomy because of the limited availability of experts prepared to contribute sufficient time to its maintenance, although using an open platform such as GitHub to manage the GBIF Backbone Taxonomy (Page 2013) may ease the limitation, in comparison with the Catalogue of Life approach. Even if such a unified, single taxonomy were available, it would still need to provide the capacity for a user to choose a preferred taxonomy or taxonomies from alternatives: no single taxonomy will satisfy everyone. It implies that we need to store multiple taxonomies and a method to choose, or at least compare, them by uniting those stored taxonomies without 'unitary-ism' (Berendsohn and Geoffroy 2007).

Data structures capable of storing multiple taxonomic views have been proposed (Berendsohn 1995, Pullan et al. 2000, Ytow et al. 2001, Pyle 2004) and applied to taxa of various scopes up to a nomenclatural code scale (Pyle and Michel 2008). Name services such as uBio, ZooBank, Global Names Usage Bank, Plazi and GBIF Checklist Bank are available as data sources of taxonomic views with APIs.

Software systems have been developed to compare two or more taxonomies visually from either the taxonomy or the data-visualisation perspective (or occasionally both) (Graham and Kennedy 2010). Taxonomy-oriented systems show rich information that expert users require, but in general needs significant extra interpretation that may be beyond the skill of non-expert end-users. Displaying rich information can also result in a reduction of the manageable number of taxonomic views, especially if relationships between the views are complicated, where such software systems are most useful. Taxonaut, development of TaxoNote (Morse et al. 2003) to facilitate navigation over taxonomies, is a Java application software which is designed to provide summarised, simultaneous comparison of two or more hierarchies, focusing on the needs of non-expert users. It sticks to conventional display components, specifically a table of folder-style tree structures, rather than, for instance a set of directed acyclic graphs, to reduce novice users' difficulty in interpretation. It uses two representation methods in combination which are categorised as agglomeration and matrix respectively (Graham and Kennedy 2010). Application of the software is, however, limited by the number of available taxonomic views represented as data sets. A stable version of GBIF Species API was released recently with comprehensive Java libraries, which provides a reliable test case of multiple taxonomies comparison. Note that during preparation of this manuscript the GBIF backbone was updated (Döring 2016). Results presented here were based on the previous version of the backbone (GBIF 2013). This contribution describes the extension of Taxonaut to use the GBIF Species API to recover multiple taxonomies and reveal issues experienced in handling such 'real' data. The software is extensible to other data sources because it is designed to accept multiple data sources from different APIs.

## Materials and Methods

### Data source

GBIF ChecklistBank stores more than 2500 checklists contributed by data providers. These checklists can be categorised into two groups, a small number of large checklist and many small sets of name usages extracted from separate publications. Data are available in JSON format using Species API, which includes name usages in checklists, the higher and lower name usages (parent and child name usages), synonyms and citations. Java libraries with source code are available to use the API. Most of the name usages have been mapped to the GBIF Backbone Taxonomy, a synthetic hierarchy intended to cover all checklists in the Checklist Bank. The Backbone Taxonomy is available as one of the stored checklists.

### Design goals

Taxonomies comprise an organised list of taxonomic names that are drawn from diverse data sources and organised according to an expert in the local domain. ﻿Publication of new taxa after the publication of the main hierarchy will often, but not necessarily, specify the hierarchical position that the author intends for the new taxon. Various experts will inevitably propose a variety of such views (Ytow et al. 2001). TaxoNote (Morse et al. 2003) was built to facilitate comparison of disparate taxonomies. Taxonaut was designed to facilitate graphical exploration of the information space that these taxonomies represent.

The graphical user interface (GUI) components of Taxonaut have been modified from TaxoNote to improve usability. TaxoNote read data from local XML files in the InfoVIS2003 Contest tree format (Fekete and Plaisant 2003) where each file contained a single hierarchy. Therefore, hierarchies to be compared are specified by selecting flies to read. Taxonaut, on the other hand, gathers remote data fragments using GBIF Species API in an interactive manner described below. GUI components supporting this interaction were enhanced. See "General work flow" section for detail of this enhancement, three sub-panes named "Name usages", "Detail" and "Name Tree." Third-party software components are listed in "Availability" section.

### Ostensive comparison of taxon concepts

A formal model is required to establish the equivalence of taxa. A taxon is a named concept that has been represented as a set of individual organisms (e.g. Gregg 1954, Geoffroy and Berendsohn 2003). Such sets cannot be treated formally as mathematical sets (Ytow et al. 2001) but can be handled as rough sets (Pawlak 1991). The plasticity of taxon concepts, which are intrinsically open sets, means that strict application of set equivalence is not practically useful, being highly sensitive to set completeness. Each taxon can be represented ostensively by some individuals or lower taxa and rough set theory used to establish equivalence (Ytow et al. 2006). Note that a lower taxon is not a member but a subset of the higher taxon.

Rephrasing in terms of taxonomy, a taxon concept can be represented by aggregations of lower taxa that are included and excluded. Name usages in databases, also known as potential taxon (Berendsohn 1995), Nomenclatural Taxon (Pullan et al. 2000), Name Record (Ytow et al. 2001), Assertion (Pyle 2004), TaxonConcept (Taxonomic Names and Concepts interest group, Biodiversity Information Standards (TDWG) 2005), or TaxonNameUsage (Pyle 2016), provide these taxon concepts as building blocks. This approximation enables the evaluation of ostensive compatibility of taxon concepts instead of exhaustive set equivalence.

### Alignment of taxa on screen

**Figure 1.**
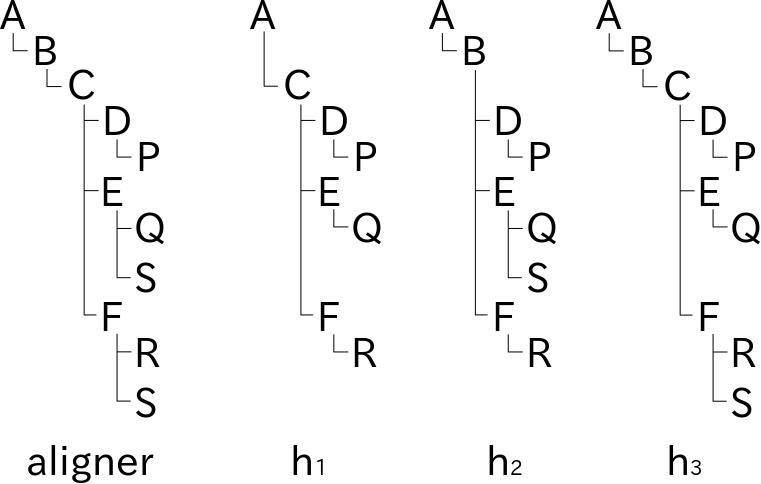
Schematic explanation of aligned rendering of name usages in hierarchies on screen. Name usages in three tree diagrams representing hierarchies, h_1_, h_2_ and h_3_ are aligned with the aid of an aligner tree. Capital letters in hierarchies represent name usages in each hierarchy. The aligner tree is composed from hierarchies as a kind of superset where letters in the aligner tree are place holders to determine vertical offset of nodes. Rendering of a tree diagram in two dimensions based on the folder metaphor requires calculation of node position where horizontal offset depends on depth of the node while vertical offset is relevant to both depth and width. Nodes in hierarchy diagrams can be aligned horizontally by sharing the calculation of vertical offset of nodes. For example, vertical position of node C in hierarchy diagram h_1_ is determined by vertical offset of node C of the aligner tree while horizontal offset is determined in the diagram h_1_ alone. Inconsistency between hierarchies results in duplicated nodes in the aligner tree; different assignments of name usage S in hierarchies h_2_ and h_3_ results in two S nodes in the aligner tree. Vertical offset of nodes S in hierarchies h_2_ and h_3_ are determined by position of corresponding nodes S in the aligner tree. Missing name usages, e.g. name usages B and S in hierarchy h_1_, result in vacancies in the hierarchy diagrams like vacancy for boundary sets of Rough set representation. Note that such vacancies do not affect the horizontal offset in hierarchy diagrams.

There are ways to show relatedness of taxa displayed on screen (Graham and Kennedy 2010), such as linking taxa by a line (Craig and Kennedy 2008), highlight taxa by shared colours, or overlapping by animation. Taxonaut uses alignment of taxa for this purpose, using the two dimensions of the screen in a consistent manner, one for multiple hierarchies, the other for taxa in each hierarchy. This is visually similar to the familiar tree structure used to display files in a computer. For operational consistency, equivalent name usages in column are expected to be aligned horizontally. A composite tree structure, a tree not in sense of phylogeny but graph theory, covering all name usages in hierarchies under comparison is used to align name usages in each hierarchy (Fig. 1). Thus names appear on an appropriate line with an indentation representing the depth of the node. Vertical placement of name usage in a hierarchy under comparison is determined by the composite tree while horizontal placement is decided by the hierarchy. Alignment by the composite tree is robust to missing name usages in each hierarchy which appear as gaps in each hierarchy. If name usages of the same name literal in hierarchies have incompatible paths to the tree root, the composite tree has two or more nodes of the name (e.g. taxon S in the aligner tree in Fig. 1). Note that the composite tree will not provide a taxonomically sensible hierarchy if hierarchies under comparison are inconsistent with each other.

### Implementation of composite tree

**Figure 2.**
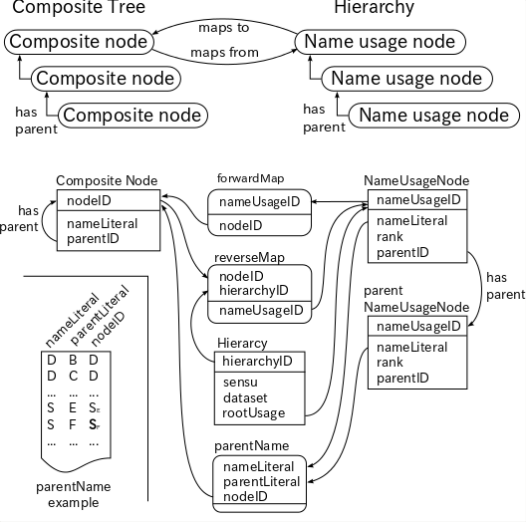
Schematic diagram of the data structure for the composite tree. In addition to the fundamental TreeModel data the composite tree possesses three mapping tables. First the forward map, which links each name usage node to a composite node. Second the reverse map, which links the composite node to each name usage node in the hierarchies under comparison. It can link a single composite node to multiple name usage nodes. Third the parental name, which links pair of the name literal and its parental name literal of a name usage node to the composite node. It providers a working cache of candidate composite nodes to which the name usage node may map to. If the parental name table contains a composite node that matches a name literal and parental name literal of the name usage node in the hierarchy being processed, the composite node is a good candidate for a mapping. Examples of some composite nodes in Fig. 1 are shown in the insertion. There are two entries for composite node D, one is indexed by literals D and B while the other by D and C. The D-B indexed entry is created for node D in hierarchy h_1_, while D-C indexed entry is created for node D in hierarchy h_2_. Note that the composite tree does not retain the direct parent-child relationship between B and D after insertion of composite node C. The parental name table has this information even after insertion of composite node C to provide a quick look up table to find composite node D as a candidate for hierarchy node with D and B literal pair, otherwise it is necessary to scan the parental path against hierarchies already incorporated. The parental name table has two entries for composite nodes S because there are two composite nodes, distinguished by suffixes. The parental name table also provides a distinction between these two composite nodes. The three tables are implemented by Java HashMaps.

The composite tree is the key component for an aligned display. It is implemented by extending the Java DefaultTreeModel, a data model of JTree from the standard Java library. JTree is a GUI component to display a tree structure on screen, of which nodes can be expanded or collapsed to show or hide child nodes if present. The composite tree data is generated by integrating hierarchies in sequence, where each hierarchy is also represented by a Java TreeModel as a structural copy of the original hierarchy (Fig. 2). The first hierarchy in a group to be compared is copied directly to make the initial composite tree. Subsequent hierarchies are embedded into the composite tree as follows:

The tree representing a hierarchy is scanned by breadth-first search with a pre-fetch of one depth. The root node of composite tree is used as the parental composite node at the beginning.

- 1. Each node in the hierarchy is examined against the forward mapping to find a composite node.
  - 1.1 If found, the composite node is used as the parental node in the later steps.
  - 1.2 If not, look up the double indexed mapping by the name literal of the node. Note that it happens only on the root node of the hierarchy because of later pre-fetch.
    - 1.2.1 If there is no entry of the literal, create a new composite node as a child node of the parental composite node, add map entries between the hierarchy node and the created composite node. The composite node is used as the parental node in the later steps.
    - 1.2.2 If there is an entry of the literal that shares a parental literal, examine compatibility between parental paths of the hierarchy node against each composite node in the list. If there is only one composite node having matched path, the composite node is used as the parental composite node in the later steps. Else, create a new composite node as a child node of the parental composite node, add to maps. The composite node is used as the parental node in the later steps.
    - 1.2.3 Else, i.e. there is an entry of the literal but no composite node shares the parental literal, examine compatibility between parental paths of the hierarchy node and the parental composite node of each composite node in the list. If there is only one composite node having matched path, the composite node is used as the parental composite node in the later steps. Else, create a new composite node as a child node of the parental composite node, add to maps. The composite node is used as the composite node in the later steps.
- 2. If the hierarchy node has child node, examine them as follows
  - 2.1 Examine redundancy of name literals in child nodes of the hierarchy node. If there are 'redundant' name usages, i.e. two or more name usages of the same name literal, merge them into a single child node of the parental hierarchy node. Other redundant node should be removed after transfer of all lower nodes.
  - 2.2 Examine each child of the parental composite node against child hierarchy node, testing whether it shares the name literal. If they have the same name literal, add the hierarchy node and the composite node to maps, and rearrange the sequence of child hierarchy node to match to the sequence of child composite nodes for graphical alignment.
  - 2.3 Map each of remaining child hierarchy node to a composite node using the procedure described under 1.1 and 1.2.
- 3. Go to step 1 to process the next hierarchy node.

The algorithm is designed to be modest and conservative which maps a hierarchy node to a composite node only when the candidate composite node is unique. It might be improved by choosing a best fit composite node from two or more candidates, but it requires further investigation. Step 2.1, on the other hand, reduces multiplication in an aggressive way to reduce the burden of later matching of candidate nodes. Examples of such redundancy to be omitted will be described later. If it is necessary to compare these 'redundant' name usages, users are advised to set height of the tree to zero to cut higher taxa path.

### Availability

The source code and the executable jar file are available from GitHub, https://github.com/nomencurator/taxonaut. The executable jar file runs in Java 8 envirionment available for Linux, Mac OS X, Solaris and Windows. Instruction is available on its Wiki page. The software is distributed under the Apache Licence. Executable jar file contains following third party software: GBIF API client Java code, Apache axis including jaxrpc, Jackson version 1, Apache POI, Google guava and Find Bugs. These third party software are copyrighted by the original authors. All but Find Bugs are licensed under the Apache Licence. Find Bugs is licensed under the Lesser GNU Public License.

## Results

### General work flow

**Figure 3.**
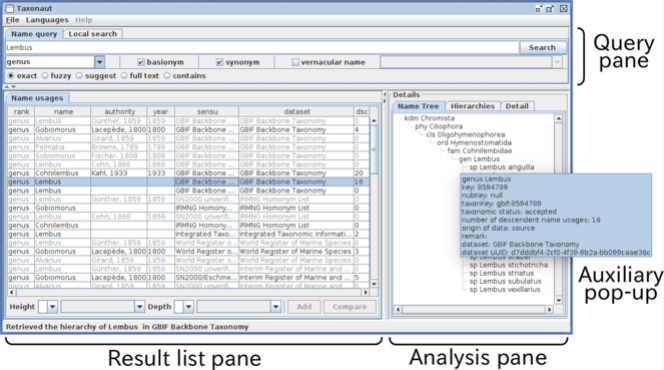
A screen shot of the software showing a result of search for *Lembus* with the basionym and synonym options. The window is divided into three panes: the Query pane, to enter query; the Result list pane to display the result of query and to select a subset of the result to display on the Analysis pane. These panes are re-sizeable by moving or clicking separators between panes. Tabs in the analysis pane enable switching between facets of the analysis.

The software display is divided into three resizable areas: Query pane; Result list pane; and Analysis pane (Fig. 3). Users are expected to enter a name or names in the query pane to get search results in the result list pane as a list. The result list is used to select records to be analysed in the analysis pane. These three panes are designed to hold sub-panes accessed by tabs allowing various operations or data types to be displayed. The current implementation, for example, has three tabbed sub-panes in the analysis pane to display taxonomic comparison, a hierarchy containing the selected name usage or detail of the name usage. These three sub-panes provide facets of analysis, with auxiliary information available in optional pop-up windows to reduce number of tabs. A new facet, e.g. a distribution map of the taxon, could be added to the analysis pane with an additional tab label. Search results other than name usage, such as publication or author, could be hosted by the result list pane as sub-panes with tabs labelled “Publication” or “Author” depending on query result available from data sources. ZooBank is an example of data sources with APIs for Author and Publication, to which connection remains to be developed. The result list pane can be used to specify target of the query, such as “retrieve publications containing this name.” The query pane can similarly handle different type of queries through tabbed sub-panes, such as bibliographic query to find information relevant to specific publication, or personal name to find data relevant to a person. These extensions are dependent on the data sources to be used and future implementation.

The current implementation has two query sub-panes to examine either Species API or local search to highlight names in the Hierarchy sub-pane described later. Users can compose a query to external data sources by name or names separated by '|', with rank of interest specified, if necessary. There are options to include basionyms or synonyms, and choice of name matching mode. If the query is a vernacular name, users can specify the language. The result of a query is accumulated into a cache to reduce access to the data source.

**Figure 4.**
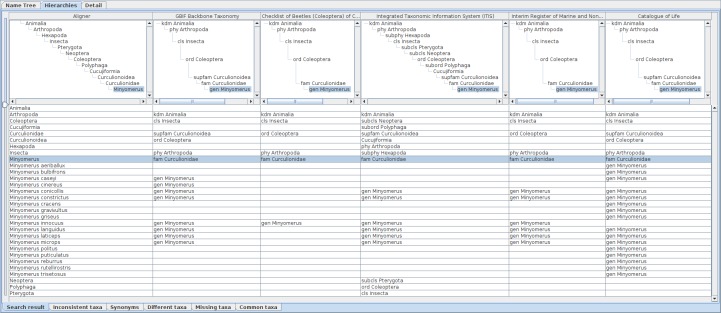
Comparative display of five datasets containing *Minyomerus* records with an aligner. Each column shows the aligner or one of the datasets as both hierarchy and name usage assignment to higher name usage. Widths of the columns were adjusted to show full higher taxonomy. Hierarchies are expanded from root node to name usages selected in the name list pane of which nodes in the tree diagrams are highlighted. A row of the table indicates distribution of the name literal in the datasets, where each cell of the aligner column contains the name literal of interest, while each cell of datasets contains higher name usages of the name literal if the name literal is used in the dataset. Therefore, missing names result in a vacancy in trees and table cells. Name usages selected in the name list pane are also highlighted in the table. Users can rearrange columns by drag-and-drop on screen depending on their interest, especially for ease of comparison. Name literals in the aligner table are sorted alphabetically.

The Name usages sub-pane in the result list pane shows name data returned from Species API in tabular format, showing information that might be useful in choosing records of interest. This sub-pane has sets of selection-boxes at the bottom to specify highest rank (height) and lowest rank (depth) of the hierarchies to be retrieved. Height and depth can be specified either as a number (e.g. 4, meaning hierarchical ranks) or name of a rank (e.g. order). Note that if both number and name are specified, the software will represent whichever is the fewer intermediate ranks, because some taxonomies utilise large numbers of intermediate ranks between the widely recognised major ranks (e.g. class, order, family). By default the software will recover the highest and deepest records available from the recovered data. By selecting a line in the list, the software will show details of the record in the “Detail” sub-pane in the Analysis pane, and retrieve the hierarchy of specified height and depth to show in the “Name Tree” sub-pane. Selection of two or more lines enables the button also at bottom of the pane to compare selected name usages. The button triggers retrieval of hierarchies containing each name usage selected and their display in a comparison table of the “Hierarchies” sub-pane in the Analysis pane. Since sub-panes in the Analysis pane are independent of one another, users can examine the detail of a hierarchy under comparison by selecting a line in the Name usage sub-pane. Hierarchies sub-pane is described previously (Morse et al. 2003) as hierarchy comparison panel and assignment table. The Hierarchy sub-pane (Fig. 4) contains a set of tabbed panes each of which contains a table to display the result of comparative analysis. The Search Result tab contains all name usages in all hierarchies, while the other tabs, Inconsistent taxa, Synonyms, Different taxa, Missing taxa, and Common taxa, contain subsets of name usages that fall in these categories. Each column of these tables represents a hierarchy, or the composite tree in the leftmost column. Users can reorganise and resize columns. The table is divided into upper and lower parts with a slider to resize these areas. The height of the upper part is shared by tables of the other tabs. The upper part shows hierarchies and the composite tree in graphical structure. Positions of nodes in hierarchies are calculated as described above. Expansion or collapse of a node in a hierarchy or composite tree is synchronised with that of corresponding nodes in other hierarchies or composite tree. Expansion/collapse of nodes are synchronised between tables of different tabs of the hierarchy sub-pane. Vertical and horizontal scroll bars accompanying each tree graphic allow users to display area of interest. The lower part of the table is explained in the following sections with examples.

Because of volume and diversity of available data, it is impractical to show the result of an exhaustive search. Some example tasks are described instead.

### A rather simple example

**Figure 5.**
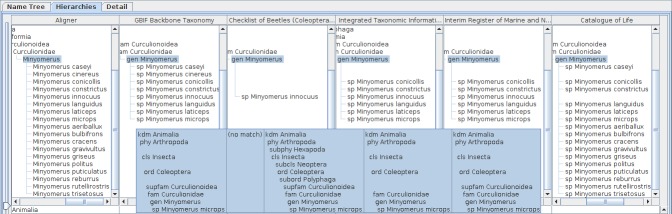
Comparison of *Minyomerus* species in hierarchical trees. Hierarchical display of member taxa uses a screen more efficiently than that of higher taxa path, because the latter results in sparse screen containing diagonal name path. Although a taxon concept is represented by a pair of intent and extent represented by higher taxonomy and member taxa, respectively, it is better to focus on either of them at once on a screen because of the nature of tree diagrams. Users can adjust what is shown on the display by scrolling hierarchies and resizing the display area to focus on their interest, e.g. showing membership hierarchies only. An auxiliary window pops up by placing cursor (not shown for clarity) on a name usage which gives summary of higher taxa path.

Recent revision of *Minyomerus* (Insecta: Coleoptera) (Jansen and Franz 2015) with aid of Euler/X (Franz et al. 2015), software to compare two taxonomies, gives a visual summary of its revision history. Query of *Minyomerus* returns five records of five data sources, GBIF Backbone Taxonomy, Integrated Taxonomic Information System (ITIS), Interim Register of Marine and Nonmarine Genera (IRMNG), Catalogue of Life, and Checklist of Beetles (Coleoptera) of Canada and Alaska, Second Edition. Comparison of those records gives five columns and an aligner column after integration mentioned above (Fig. 4). The moderate height of higher name usages paths enables the display of all hierarchies in a display of ordinary size. Numbers of species names under *Minyomerus* vary with data sources (Fig. 5), from one for Checklist of Beetles to 16 for Catalogue of Life covering almost all the species names that appeared in (Jansen and Franz 2015), except *Minyomerus imberbus* which is newly circumscribed in the revision. ITIS and IRMNG share the same six species names which are members of GBIF Backbone Taxonomy. Catalogue of Life does not contain *Minyomerus cinereus* found in the GBIF Backbone Taxonomy nor *Minyomerus innocuus* which is contained in the other data sources. Aligned display of name usages, either in hierarchies or in the name table, enables a comprehensive comparison between taxonomic views.

**Figure 6.**
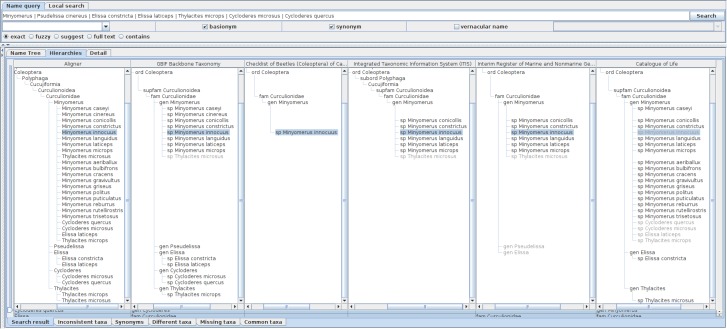
A screen shot showing hierarchical comparison of a union query of *Minyomerus* and relevant species names including their basionyms and synonyms. Name usages declared as synonyms in data sources are displayed in shaded characters. Union query of *Minyomerus* and relevant species names including their basionyms and synonyms returns extra records which are unavailable without these extensions. For example, Catalogue of Life returns *Minyomerus innocuus* which is not included in the child name usage set of *Minyomerus* although its higher name usage is specified as the *Minyomerus* usage. It is retained as a synonym of *Minyomerus microps*, which suggests that *Minyomerus innocuus* is not included in the child name usages because it is not an accepted name but the relationship to the higher name usage is recoverable via its accepted name, *Minyomerus microps*. The *Minyomerus innocuus* in Catalogue of Life was recovered by recursive query of synonyms through *Minyomerus microps* and *Thylacites microps* which is specified as a part of the union query. There are other synonyms of *Minyomerus microps* shown under the *Minyomerus* usage of Catalogue of Life.

Extending the query to include basionyms and synonyms by specifying these options enables the examination of the data sources that incorporate the synonym treatments. The genus level query can be further expanded by adding species names relevant to those synonymous species to the query enables the inclusion of those 'illegitimate' name usages as siblings of other ordinary usages under the higher name usage (Fig. 6).

**Figure 7.**
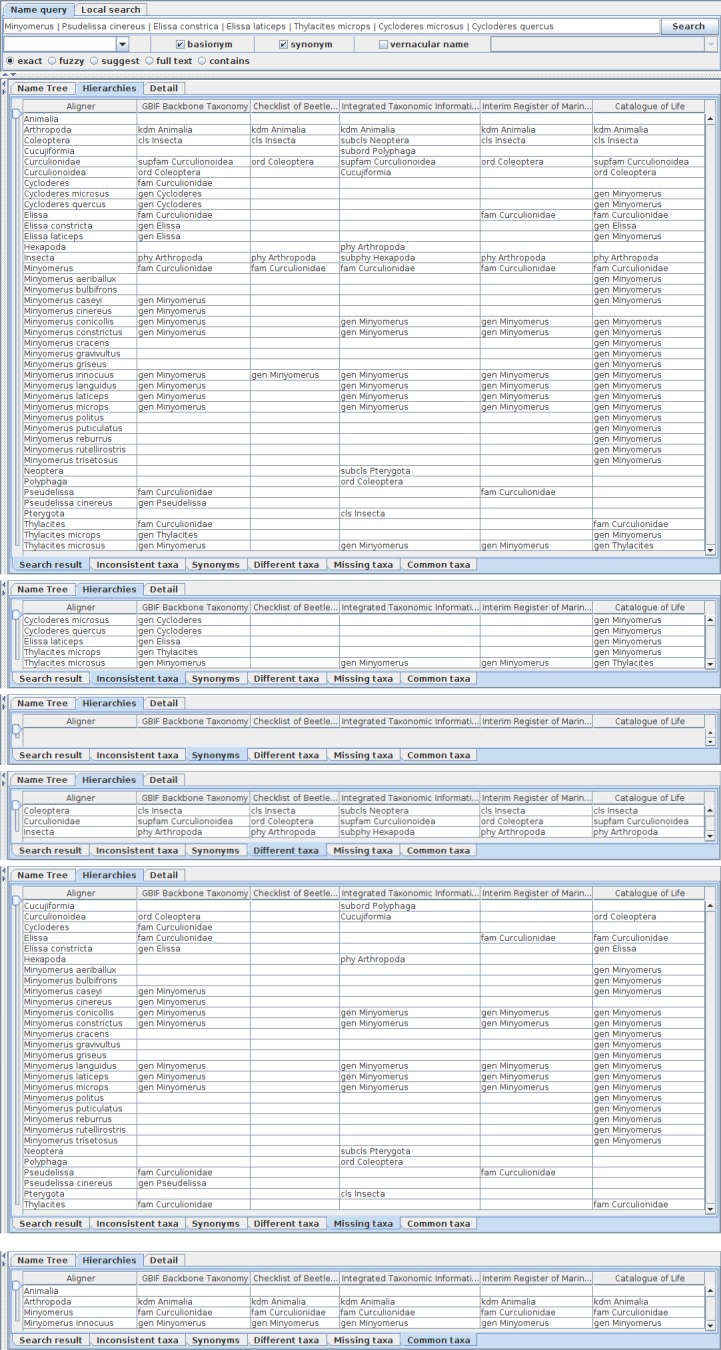
Facets of assignments to higher name usages are shown in assignment tables with tabs of facet names where the word 'taxa' is used for 'name usages' to save screen space. Five hierarchies obtained by the union query are the same as in Fig. 6, are shown in both tree diagrams and assignment table where a row represents assignments of name usages having the name literal in the aligner column to higher name usages in hierarchies, although tree diagrams are minimised in the figure for clarity. Assignments are categorised into facets which can be switched by selecting tabs at bottom of the table. Note that names in table cells of hierarchies represent higher name usages to which the name literal of the row is assigned, but not name usages of the name literal. Rows are ordered by the name literal alphabetically for easier looking up. The table of 'Search result' tab shows distribution of all assignments of name usages. The literal of name usages at the root of hierarchies, i.e. Animalia in this case, is shown without higher name in any hierarchy. The table of 'Inconsistent taxa' tab shows inconsistent name usage as different higher names of the same lower literal. It does not contain, however, assignments that differ only in intermediate name usages, which can be embedded into the other assignment. Such higher-name-path conformal assignments are categorised into the table of 'Different taxa' tab which shows compatible but somewhat different assignments. The synonyms tab would cover synonyms between hierarchies, rather than synonyms given in each hierarchy, remains to be implemented. The table of 'Missing taxa' shows name usages that appear in some, but not all, hierarchies without inconsistency. A vacant cell indicates that the name literal does not appear in the hierarchy. The table of 'Common taxa' shows name usages that shares both its literal and higher literal in all hierarchies.

The lower part of the table shows a distribution of name usages in hierarchies with categorisation based on analysis during composition of the aligner tree (Fig. 7), i.e. name usages that appeared in all hierarchies sharing the same name literal and higher name usages, or different in higher name usage, name usages missing in some hierarchies, inconsistent name usages, synonyms, and all name usages. Each cell contains the higher name usage of the name literal on the line. Inconsistent name usages are extracted from the composite tree alone, because it retains nodes covering all name usages for the alignment and hence inconsistencies, both inter- and intra-hierarchies, are reproduced in the aligner tree. Synonym tab is designed to show name usages having different higher names depending on taxonomic views where higher names are not on the path of higher nodes of the composite tree. Synonymous usages also appear in the 'different taxa' tab because they are different in their higher name literal. Note that the synonym tab is expected to capture potential synonyms between hierarchies rather than what is stated as a synonym in a single hierarchy.

This example allows an informed user to reconstruct the taxonomic history of the genus as well as assessing the differences in coverage of the various data sources. In this case, the nomenclatural history of the genus has been expertly assessed by Jansen and Franz (2015) and the interested reader can follow the changes to the nomenclature. The objective of the software, however, is to provide rapid access to these changes so than an expert can more quickly undertake a new revision of a group less well studied. The non-expert user can more readily judge which hierarchies are more useful for their own purpose.

### Higher name usages

Query of *Lipotes* returns 13 genus usages from distinct data sources, where each of them assigns the genus to one of the Cetacea (Mammalia) families, Iniidae, Lipotidae or Planistidae. Five of 13 data sources, GBIF Backbone Taxonomy, Mammal Species of the World, Integrated Taxonomic Information System (ITIS), Interim Register of Marine and Nonmarine Genera, and Catalogue of Life assign *Lipotes* to Iniidae; three data sources, Taxon list of animals with German names (worldwide) compiled at the SMNS, Phthiraptera.info and Catalogue of Life China assign *Lipotes* to Planistidae; and the remaining five, Paleobiology Database, NCBI Taxonomy, World Register of Marine Species and Wikipedia Species pages of both English and German assign it to Lipotidae. Interestingly, neither Global Names Usage Bank nor ZooBank retains a *Lipotes* record, even though *Lipotes vexillifer* is on the IUCN Red List where the genus is assigned to Iniidae with remark saying that it was assigned to Planistidae in the previous Red List.

**Figure 8.**
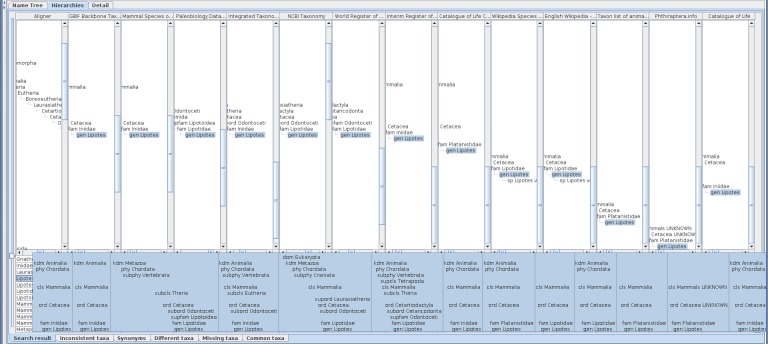
Hierarchies containing *Lipotes* name usage are compared on a screen of 1920 pixels wide. Width of each column is insufficient to display a hierarchy of full depth but the pop-up gives a sketch of hierarchies where unranked name usages are not displayed.

Choosing those 13 name usages gives a table of the hierarchies with an additional composite tree to align name usages in each hierarchy (Fig. 8). Users can adjust the view port and width of each column to display the area of interest. Arrangement of columns follows the order of hierarchy evaluation which is determined by the order of selected name usages in the list. The user can rearrange the columns for easier comparison between hierarchies without affecting the composite tree structure.

**Figure 9.**
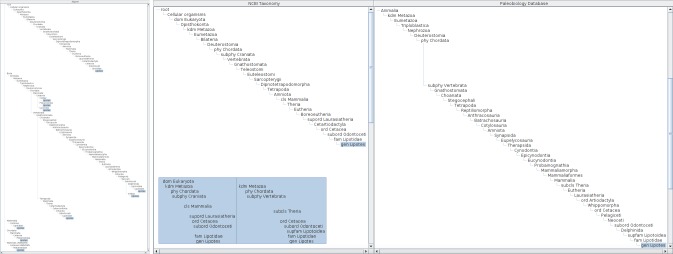
Two columns of the assignment table are cut out to compare hierarchies of long higher name usages path each other, with an inserted pop-up screen showing their summary. Full aligner tree covering the result of *Lipotes* query is also shown on the left of these columns. The result hierarchies of *Lipotes* query contain two hierarchies with long higher name usages paths which result in two independent major branches in the aligner tree making alignment of hierarchies difficult. Most of name usages in those long paths are unranked which do not appear in the pop-up summary.

**Figure 10.**
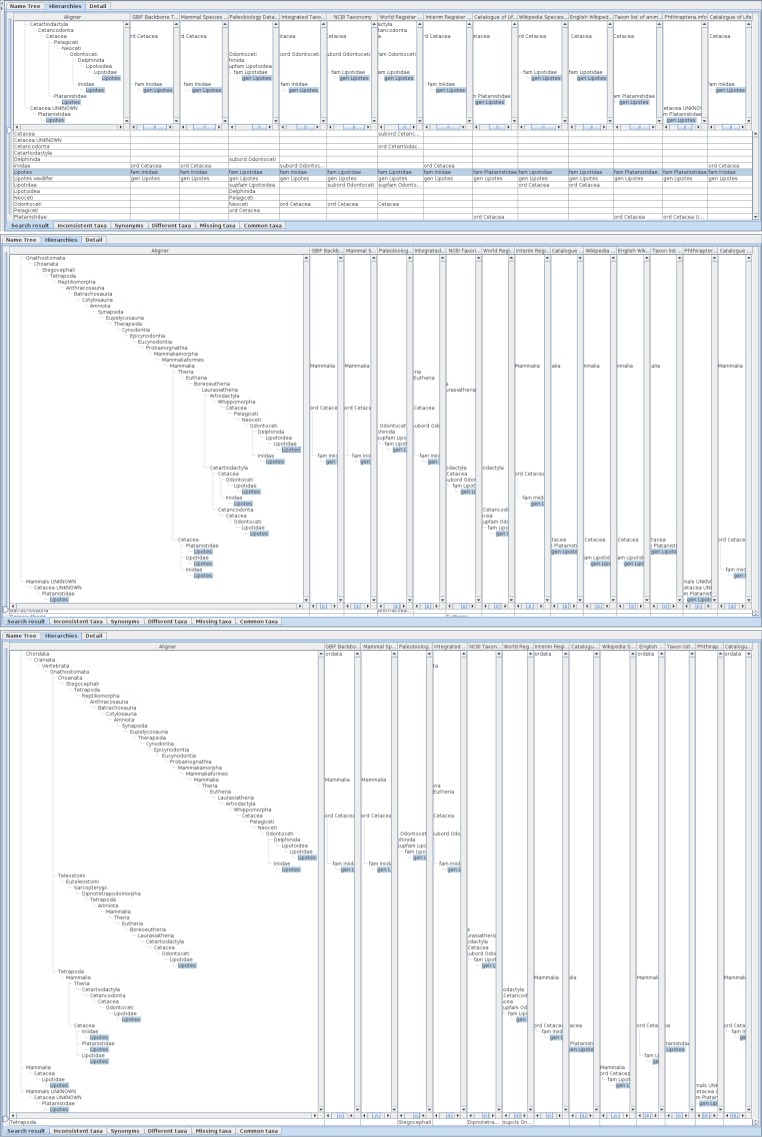
Alignments with different height of hierarchies containing *Lipotes*, up to order (upper), class (middle) or phylum (bottom), respectively. The height and depth of hierarchies being compared can be specified in the name usage list pane by either rank or number of levels above and below the name usage. If specified by rank, the software composes hierarchies to be limited within less than the next rank. For example, if class is specified as upper limit of hierarchies, the results may contain name usages less than phylum to cope with unranked intermediate name usages. The aligner tree for hierarchies up to order has four branches containing *Lipotes*. One branch is an artefact of misinterpreted data, Cetacea UNKNOWN, where UNKNOWN should be processed as authority rather than a part of name literal itself. Other three branches are results of different family names which require independent path between order and genus. The aligner tree for hierarchies up to class contains 9 *Lipotes* branches. Besides the erroneous UNKNOWN branch, remaining eight branches are joined into three higher branches depending on intermediate taxa between Mammalia and Cetacea, i.e. without intermediates, via Artiodactyla or Cetartiodactyla. Note that hierarchies without intermediates are mapped to branches in aligner tree either with or without intermediates depending on the sequence of integration into the aligner tree, because addition of an inconsistent path to the aligner tree requires the choice of one of those paths to which a new direct hierarchy is mapped but there is no way to choose one of them. The aligner tree up to phylum also contains 9 branches of *Lipotes* including one erroneous 'UNKNOWN'. Although the number of branches is the same to that for class, branching points get higher.

The alignment algorithm is sensitive to higher classification because it uses top-down name matching. Amongst those 13 hierarchies, Paleobiology Database and NCBI Taxonomy use higher, or, more intermediate taxa in their classification than others. The highest taxon in most of those classifications is either Animalia or Mammalia except “root” of NCBI Taxonomy and Biota of World Register of Marine Species which are higher than Animalia and incompatible each other in their name. Those two highest names results in two independent trees (Fig. 9) which can be partially unified by limiting the highest level of taxon (Fig. 10). There are two incompatible hierarchy paths between Mammalia and Cetacea which results in two Cetacea nodes in the composite tree. Detail of the composite tree can depend on the sequence of incorporation of hierarchies into the tree.

### Data inflation in higher name usages

Berendsohn (1995) pointed out that "a database system based on potential taxa is open to an inflation of records: any name referred to in a publication may form a new potential taxon". GBIF ChecklistBank is prone to such data inflation.

Search of *Inia*, a genus of Iniidae (Mammalia: Cetacea), returns 20 name usages where each of NCBI Taxonomy, Catalogue of Life and German Wikipedia Species Pages returns two records and English Wikipedia Species page returns three records. Other data sources contain a single record for each. Both records of NCBI Taxonomy were derived from the original data source. One of them without rank is said to be a synonym of *Inia* with genus rank. One of the records attributed to Catalogue of Life was created at GBIF from a de-normalised classification, assigned to Animalia. Both records attributed to German Wikipedia are records from de-normalised classifications of different height; one up to order Cetacea while the other up to class Mammalia. Each of two *Inia* records has only one species, either of *Inia geoffrensis* or *Inia araguaiaensis*, where these data originate in German Wikipedia. It implies that these two species data in German Wikipedia have different higher taxa. All higher name usages of *Inia araguaiaensis* are marked as from de-normalised classification, while Mammalia and Cetacea records of *Inia geoffrensis* came from German Wikipedia. Difference in highest name usages in the data source might be the cause of those unshared Cetacea records. One of three *Inia* records attributed to English Wikipedia Species page is genuine and the other two records are from de-normalised classification. Each of these *Inia* records has one lower name usage, either of *Inia araguaiaensis*, *Inia geoffrensis* or *Inia geoffrensis boliviensis*. Via family Iniidae, the last one assigned to order Artiodactyla, while others are assigned to order Cetacea. These three subgenus records share the kingdom record, the latter two shares a phylum record, but records of class or lower are unshared. Records of higher name usages originated in the data source directly are kingdom Animalia of the three, phylum Chordata and genus *Inia* of *Inia araguaiaensis*, and class Mammalia of *Inia geoffrensis boliviensis*. All other name usages of genus or above have been created as a consequence of data processing by the aggregator.

Search of Iniidae, the family containing *Inia*, returns three records attributed to German Wikipedia and five records attributed to English Wikipedia, and each a single record for each data source. Search of Cetacea returns five records attributed to German Wikipedia and 7 records attributed to English Wikipedia. Search of Artiodactyla also returns 7 records attributed to English Wikipedia. Search for Mammalia returns 3 records attributed to German Wikipedia and 11 records attributed to English Wikipedia. Search for Chordata returns two records attributed to German Wikipedia and four records attributed to English Wikipedia, two of latter have 21,998 and 47,985 descendent name usages, respectively. This is an example of the type of data inflation mentioned in Berendsohn (1995). While Wikipedia could be the worst case of data inflation, multiple name usages in a single data source is not necessarily data inflation if the data source is expected to retain taxonomic views. Unification of records that look like replicates should remain with application software and end users because its appropriateness depends on nature of data sources and purpose of data usage.

### Homonyms and synonyms

A genus of fish was named as *Lembus* by Günther in 1859. The same literal *Lembus* was assigned to a genus of protozoa by Cohn in 1866. Kahl renamed the protozoan genus as *Cohnilembus* in 1933. By assignment of the fish species *Lembus maculatus* to another genus (currently *Gobiomorus*), the monospecific genus *Lembus* disappeared and the name literal *Lembus* became unavailable. Species of protozoan *Lembus* are now classified into three genera, *Cohnilembus*, *Kahlilembus* and *Pesudocohnilembus*. Ten other genera are relevant to the protozoan *Lembus* because some species were moved to *Lembus* from *Anophrys*, *Cyclidium*, *Lembadionella*, *Lembadion*, *Philasterides*, *Proboscella*, *Sparotricha*, *Trichonema*, *Uronema* and *Vibrio*. Retrieval of data relevant to those genera was examined, by specifying search options. Taxonaut has the following name search options, three checkboxes to include basionyms, synonyms and vernacular name, and a set of radio buttons to choose one of matching modes, exact, fuzzy, suggest, full text or contain. The ‘contain’ option is slightly different from other matching mode options. It returns name usages containing name usage of the query literal, or a list of higher taxa in essence.

The default search mode is exact matching. It retrieves 15 records, two from GBIF Backbone Taxonomy, two from Interim Register of Marine and Nonmarine Genera, two from IRMNG Homonym List, one from Integrated Taxonomic Information System (ITIS), one from World Register of Marine Species, 4 from Global Name Usage Bank, Catalogue of Life and one from Catalogue of Life 2013 Annual Checklist. Interim Register of Marine and Nonmarine Genera and IRMNG Homonym List contain both protozoan and fish *Lembus* which originate in Nomenclator Zoologicus. GBIF Backbone Taxonomy also covers both *Lembus*, protozoa from Catalogue of Life and pisces from World Register of Marine Species. All four records from Global Name Usage Bank represent fish *Lembus*. Matching options other than ‘contain’ returns the same set of name usages returned without options.

The Name list pane shows synonyms in shaded text for easier distinction from accepted names. Selecting a line of shaded text, the Detail pane shows information including the accepted name. Amongst of those 15 records, ITIS, CoL and one of GBIF Backbone Taxonomy data are claimed as valid names. Three of 4 records from Global Name Usage Bank are claimed as synonym while the other is stated as doubtful. Others give their accepted name, either *Cohnilembus* or *Gobiomorus*, except one record from Global Name Usage Bank which claims that *Lembus* is valid name of synonym *Lembus*. Those synonym records from Global Name Usage Bank originate in different works, 'Eshcmeyer (2004)', 'Eshcmeyer (1998)' and 'Günther (1859)', which results in the record stating that valid *Lembus* is synonym of *Lembus*. Besides this self synonymy, multiple name usages of the same name literal is in the nature of the design because Global Name Usage Bank provides access to accumulated name usages instead of giving a single consensus view unlike CoL or GBIF Backbone Taxonomy. Note that one of two records of CoL saying 'denormed classification' is not an original one but was created at GBIF which places it at just under kingdom, while the other is placed as a member of a family.

There are two possible ways to expand the query to include synonyms *Cohnilembus* or *Gobiomorus*, by adding these known synonyms to the query, or use options basionym or synonym. Because *Lembus* is a basionym of *Cohnilembus* but not of *Gobiomorus*, basionym option returns *Lembus* and *Cohnilembus*. The ‘synonym’ option can also recover more than *Gobiomorus* by recursive synonym query of Taxonaut. 'Lembus or Cohnilembus' query returns 9 *Cohnilembus* records adding to the 15 *Lembus* records. Search of Lembus with basionym option returns the same set of records. It is interesting that CoL, CoL2013, ITIS and GBIF Backbone Taxonomy contains both *Lembus* and *Cohnilembus* as accepted names. Considering origin of these data sets, it seems to be inherited from ITIS to GBIF Backbone Taxonomy through CoL.

A query also with *Gobiomorus* results in more 16 *Gobiomorus* records from 11 data sources where two records are from CoL and 5 records from Global Name Usage Bank. Four of 11 are new data sources, English Wikipedia, Fishes of the Neotropics, NCBI Taxonomy and TAXREF. One record of CoL is marked as de-normed classification suggesting an artefact of importing into GBIF Classification Bank. Five records of Global Name Usage Bank contain two views, *sensu* Eschmeyer 1998 and Eschmeyer 2004, and a de-normed classification. Each Eschmeyer view is composed from two records, *Gobiomorus* Lacépède, 1800 with status 'synonym' (e.g. key: 108885839) and *Gobiomorus* Lacépède, 1800 with status 'doubtful' (e.g. key: 108885835) which is specified as accepted record of the former as 'Gobiomorus Lacépède, 1800 sec. Eschmeyer 1998', or 2004 in the other view. Interim Register of Marine and Nonmarine Genera contains *Philypnus macrolepis* as a member species for synonymy of genus *Philypnus*. Note that such detail of a record is shown in a pop-up display by placing the cursor on nodes in Name Tree pane without an additional search.

Query of *Lembus* with both basionym and synonym option returns 6 more *Alvarius* records from 4 data sources, 4 *Pelmatia* records from 3 data sources, 8 *Phylypnus* records from 5 data sources and two *Sobiomorus* records from two data sources. It doesn't cover genera relevant to protozoan *Lembus* species except *Cohnilembus* which is synonym of the genus itself. This difference in recovery of synonyms between two genera named *Lembus* reflects quality of data sources.

### Beyond homonymy: A single name literal valid under nomenclatural codes

Each nomenclatural code is independent of other nomenclatural codes. A single name literal can be a valid name of a taxon under different nomenclatural codes, for which a term hemihomonym was proposed (Starobogatov 1991, Shipunov 2013​). *Ficus* is, for example, a valid genus name of figs under the botanical code and of a group of sea snails under the zoological code. Nomenclatural data have been independently gathered depending on nomenclatural codes covering taxa of interest, e.g. IPNI or Zoological Record. The code relevant to the names recovered is thus implicit, but can be compared with the endings used for taxa above genus level, as a possible mechanism of data validation. There are, however, too many exceptions to the standard endings for this to be more than a flag for the user’s attention. This implicit dependency on classification creates potential confusion in modern classifications and is an easily avoided source of difficulty if database compilers simply made the code under which they are working explicit. Some hierarchies are not explicitly constructed under any nomenclatural code, specifically phylogenies created from molecular data. They do not refer to kingdom in botanical/zoological sense being purely reliant on the specimen sampled. Making the explicit declaration of the relevant code under which the name is used would be of enormous value for subsequent machine processing.

**Figure 11.**
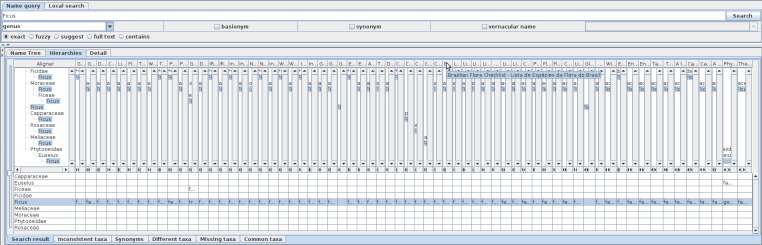
Comparison of *Ficus* usages as hierarchies up to family, without lower name usages. 65 hierarchies from separate data sources are shown with pop-up window revealing the name of data source where the cursor is placed on. Capparaceae is included as a side effect of synonymisation of a *Ficus* species to a *Capparis* species. Phytoseiidae is included as an artefact of treating the description of specimen where the name was obtained. Rosaceae and Meliaceae are included in only one data source, and hence there are two families containing *Ficus*, each for animal and plants.

**Figure 12.**
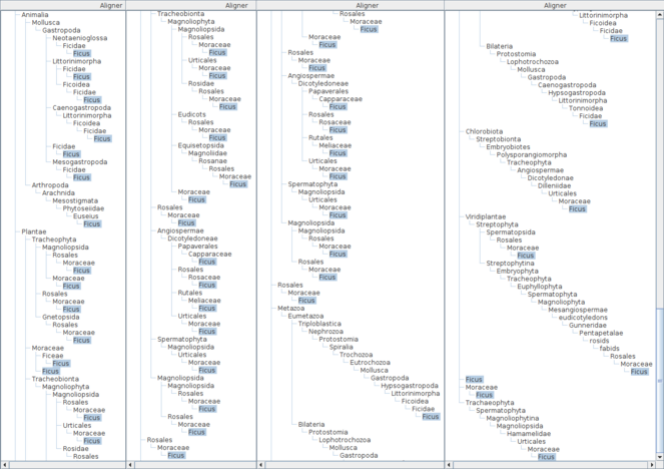
The aligner tree of *Ficus* from kingdom to genus, as a horizontal composite of four captured screens instead of vertical composition. Some branches are overlapped intentionally to enable confirmation of its coverage. There are 36 *Ficus* branches in the aligner tree for 55 hierarchies compared (not shown). Besides Animalia and Plantae, there are four major branches, starting with Metazoa, Chlorobiota, Viridiplantae and Tracheophyta, which require implicit taxonomic knowledge to evaluate homonymy.

Query of *Ficus* against GBIF Species API returns 110 records consisted of 77 generic, 4 subgeneric, two series, three specific and 24 unranked records. The unranked records contains four (presumably) cultivars, 16 species records having species epithets starting with a capital letter, and 4 records were deduced as generic records. Specifying genus as rank of *Ficus* query, the software gives 101 records as a union of 76 generic records and 25 unranked records which is the same as the unranked subset of the query without rank limitation. Comparison of hierarchies containing these generic records within ranks lower than order by selecting family in 'Height' option shows six families (Fig. 11), i.e. Ficidae, Moraceae, Capparaceae, Rosaceae , Meliaceae and Phytoseiidae, of 65 hierarchies. Endings of these family names suggest two zoological genus and four botanical genera. Capparacae appears via *Capparis membranifolia* to which *Ficus marchandii* is synonymised according to Synonymic Checklists of the Vascular Plants of the World incorporated into Catalogue of Life. Two *Ficus* records under Phytoseiidae are tricky. These records of GBIF, keys 119208195 and 119208340, of which source data are 0471C2254075FF91FEB2ACA5FD5C9100.syn3 and 0471C2254054FFB6FEB2AF6DFBC89100.syn7, are said to be synonyms of *Euseius* (Arachnida: Mesostigmata) species, *Euseius lokele* (Pritchard & Baker) and *Euseius tutsi* (Pritchard & Baker), respectively. The source data on Plazi (http://plazi.org), http://treatment.plazi.org/id/0471C225-4075-FF91-FEB2-ACA5FD5C9100 or http://treatment.plazi.org/id/0471C225-4054-FFB6-FEB2-AF6DFBC89100, say that some specimens of these *Euseius* species were on *Ficus*, presumably fruits. Rosaceae and Meliaceae are included only in Catalogue of Life China, 2013 Annual Checklist for unclear reasons. As a summary, there are substantially two families, Moraceae of fig fruit and Ficidae of fig fruit shaped mollusc. Extending the coverage of comparison to include kingdom for confirmation results in 36 family branches in the alignment tree (Fig. 12) of 55 hierarchies which is less than the number of hierarchies up to family because some data set cover both zoological and botanical branches in a single hierarchy. Some hierarchies do contain neither Animalia nor Plantae which is consequence of each source hierarchy does not extend to the 'classical' kingdoms. These clues, endings or kingdoms, are insufficient for autonomous determination whether the shared name literal is a homonym, i.e. falls in a single code.

## Discussion

Capability of the software to explore multiple taxonomic views has been limited by data availability. GBIF Species API provides wider coverage data with documented API, software library, support for developers and long-term stability including financial support, compared with predecessors such as uBio or ZooBank. It does not, however, eliminate the necessity of access to original data providers using their own API to obtain details unavailable from the GBIF Species API, or to examine interpretation of source data. Some data sources of GBIF ChecklistBank contain data derived from other data sources of GBIF ChecklistBank, with or without interpretation by non-primary provider. Name usage data are inevitably interpreted by the data provider (Ytow et al. 2001), even if the provider intends to be neutral. For example, Jansen and Franz (2015) interprets Pierce's view from 1909 as *Minyomerus* contains three species *Minyomerus innocuus*, *Minyomerus caseyi* and *Minyomerus languidus*, and *Elissa* contains two species *Elissa laticeps* and *Elissa constrica*. The work of Pierce (1909), however, contains neither *Minyomerus caseyi* nor *Elissa constrica* explicitly. It is expressed such as Minyomerus sec. Pierce 1909 which should be Minyomerus sec. Pierce 1909 sec. Jansen and Franz 2015. Access to the original data is essential for examination at this level.

The number of hierarchies that can practically be compared is limited by the size of screen, the processing speed of the host computer and the download speed of the internet connection. In addition to the length of names, the path of each tree in the comparison table increases the width of column as a function of the depth of the path. Hierarchies containing more taxa at intermediate ranks results in reduction of number of hierarchies that could be practically compared. These limitations can be reduced by limiting the height of hierarchies under comparison, although this reduction of height can affect the results. An extreme way to increase number of hierarchies displayable is to narrow each column to minimum, e.g. width of one character. Each cell of the comparison table shows higher name usage if the literal of the row appears in the hierarchy, or empty if not, as illustrated in Fig. 11. At this extreme end, the row of trees showing hierarchies can be minimised by the slider on the edge of the table. Narrowing column reduces available information about higher taxon but use of the literal in the hierarchy is still visible. Colouring cells containing name usages like the list view (Graham and Kennedy 2008) could improve visibility. Adjusting width of columns could be a bit of a cumbersome task. Table lens technique (Rao and Card 1994) could help with this task, which might also make comparison of hierarchies easier by focusing on columns of interest.

Analysis of hierarchies in the assignment table remains to be extended, especially the utilisation of relationships between name usages if given, e.g. the 'related' method of GBIF Species API. Reduction of columns showing equivalent hierarchies would be helpful in keeping the number of columns in a manageable range. It would be more important to use data from sources such as Plazi where data are provided as they appeared in each publication, rather than as a checklist-style composition. It might be necessary to redesign the user interface to display relationships between hierarchies because a relationship between hierarchies is pairwise. The display of inconsistency in the assignment table depends on the fact that the composite tree contains nodes representing all name usages in hierarchies under comparison and hence it retains inconsistency to be detected. The table under the ‘inconsistency’ tab shows that each inconsistency in the composite tree is mapped to these name usages in relevant hierarchies, which implies that some of these name usages are inconsistent. It does not mean that all name usages on the row of the table are inconsistent with each other. A relationship between name usages in different hierarchies in general can involve multiple name literals, unlike inconsistency where difference assignment of a name literal is a symptom. The display of relationships between name usages in hierarchies requires more work.

Recovery of synonymous lower name usages in the example of *Minyomerus* suggests that extending hierarchical queries to search for lower synonyms, instead of adding each lower name usages to initial query, can be rewarding. It will probably result in much slower query execution because the synonym extension requires a recursive query as follows: for each of result name usages recovered by the first query for given name literals, its synonym name usages are requested; and then name literals of result synonym are used as the next seed to obtain name usages repeatedly until the set of name literals and hence name usages are saturated. It also could raise difficulties for some users because unexpected names can appear in hierarchies. Practicality of synonym extension for lower name usages needs more investigation.

The example of *Ficus* shows that information of the nomenclatural code governing name usages is essential for handling name literals, although it is almost universally implicit. Nomenclatural code, a sort of language of the name in general, might be supported as a data element in future though, for now, it is necessary to manage it within application software. It would also enable the management of ambiregnal names where a single taxon has names valid in multiple nomenclatural codes. It is suggested that NameUsage data structure of GBIF Species API be extended to manage nomenclatural code information, either by adding a new property or expanding NameType enumeration representing type of names. The NameType distinguishes viral names from other scientific names already, and hence there is no reason to prevent adding nomenclatural codes as other name types.

Finally, the accelerating rate of species loss makes the work of taxonomists so much more urgent. Few can devote the time to regular maintenance of taxonomic name-lists, but without such lists much data is inaccessible. Clearly we need to have better software tools to explore and correct the data we do have, so that new data can be integrated into a coherent framework.

## Conclusions

The software described here allows experts to assess nomenclatural data from various sources in a way that is more time-efficient than the traditional literature-based search methods. It also facilitates comparison of data sources, the majority of which are compiled without expert taxonomic oversight.

Non-expert users can also use the software to examine and assess the quality of the data available from the large numbers of data aggregation projects that exist. Large-scale data aggregators are prone to data inflation and error propagation, because much of their data gathering is perforce automatic.

It remains a truism that data quality can only be increased by more people looking at and using the data.

## References

[B3338945] Berendsohn Walter G. (1995). The Concept of "Potential Taxa" in Databases. Taxon.

[B3338955] Berendsohn Walter, Geoffroy Marc (2007). Networking Taxonomic Concepts — Uniting without ‘Unitary-ism’. Systematics Association Special Volumes.

[B3339320] Craig Paul, Kennedy Jessie, Börner Katy, Gröhn Matti T., Park Jinah, Roberts Jonathan C. (2008). Concept Relationship Editor: A visual interface to support the assertion of synonymy relationships between taxonomic classifications. Visualization and Data Analysis 2008, Proceedings of the SPIE.

[B3401237] Darwin Core Task Group Biodiversity Information Standards (TDWG) (2009). Darwin Core. https://github.com/tdwg/dwc/blob/dd3c61c13da1c39242a94f26c673eeba6c946bb5/xsd/tdwg_dwc_simple.xsd.

[B3338979] Döring M. , Updating the GBIF Backbone. http://gbif.blogspot.jp/2016/04/updating-gbif-backbone.html.

[B3406031] Fekete Jean-Daniel, Plaisant Catherine (2003). InfoVis 2003 Contest Data Format. http://www.cs.umd.edu/hcil/iv03contest/index.shtml.

[B3339025] Franz Nico M., Chen Mingmin, Yu Shizhuo, Kianmajd Parisa, Bowers Shawn, Ludäscher Bertram (2015). Reasoning over Taxonomic Change: Exploring Alignments for the Perelleschus Use Case. PLOS ONE.

[B3401317] GBIF (2013). GBIF Bakcbone Taxonomy. http://rs.gbif.org/datasets/backbone/2013-07-01/backbone-2013-07-01.zip.

[B3339047] Secretariat GBIF GBIF Backbone Taxonomy. http://www.gbif.org/dataset/d7dddbf4-2cf0-4f39-9b2a-bb099caae36c.

[B3401472] Geoffroy Mark, Berendsohn Walter G., Berendsohn Walter G. (2003). The concept problem in taxonomy: importance, components, approaches. MoReTax Handling Factual Infromation Linked to Taxonomic Concepts in Biology.

[B3339056] Graham Martin, Kennedy Jessie (2008). Proceedings of IV2008.

[B3339066] Graham Martin, Kennedy Jessie (2010). A survey of multiple tree visualisation. Information Visualization.

[B3401463] Gregg John R. (1954). The Language of Taxonomy.

[B3339076] Jansen Michael Andrew, Franz Nico M. (2015). Phylogenetic revision of Minyomerus Horn, 1876 sec. Jansen & Franz, 2015 (Coleoptera, Curculionidae) using taxonomic concept annotations and alignments. ZooKeys.

[B3339412] Morse D. R., Ytow Nozomi, Roberts D. M., Sato Akira (2003). Comparison of Multiple Taxonomic Hierarchies Using TaxoNote. Compendium of Symposium on Information Visualization.

[B3401246] Page Roderic D. M. Time to put taxonomy into GitHub. http://iphylo.blogspot.com/2013/04/time-to-put-taxonomy-into-github.html.

[B3339480] Pawlak Z (1991). Rough sets: theoretical aspects of reasoning about data.

[B3339541] Pierce W. D. (1909). Studies of North American weevils. Proceedings of the United States National Museum.

[B3339611] Pullan Martin R., Watson Mark F., Kennedy Jessie B., Raguenaud Cédric, Hyam Roger, Raguenaud Cedric (2000). The Prometheus Taxonomic Model: A Practical Approach to Representing Multiple Classifications. Taxon.

[B3401496] Pyle Richard (2016). Towards a Global Names Architecture: The future of indexing scientific names. ZooKeys.

[B3339633] Pyle R. L. (2004). Taxonomer: a relational data model for managing information relevant to taxonomic research. Phyloinformatics.

[B3339653] Pyle R. L., Michel E. (2008). ZooBank: Developing a nomenclatural tool for unifying 250 years of biological information. ZooTaxa.

[B3339665] Rao R., Card S. K. (1994). The Table Lens: Merging Graphical and Symbolic Representations in an Interactive Focus+Context Visualization for Tabular Information. Proceedings of the CHI'94: ACM Conference on Human Factors in Computing Systems.

[B3339680] Roskov Y., Abucay L., Orrell T., Nicolson D., Flann C., Bailly N., Kirk P., Bourgoin T., DeWalt R. E., Decock W., De Wever A. (2016). Species 2000 & ITIS Catalogue of Life. http://www.catalogueoflife.org/.

[B3406232] Shipunov Alexey (2013). The problem of hemihomonyms and the on-line hemihomonyms database (HHDB). Bionomina.

[B3406222] Starobogatov Ya. I. (1991). Problems in the Nomenclature of Higher Taxonomic Categories. The Bulletin of zoological nomenclature..

[B3401506] Taxonomic Names and Concepts interest group Biodiversity Information Standards (TDWG) (2005). Taxonomic Concept Transfer Schema (TCS). https://github.com/tdwg/tcs.

[B3401152] Wieczorek John, Bloom David, Guralnick Robert, Blum Stan, Döring Markus, Giovanni Renato, Robertson Tim, Vieglais David (2012). Darwin Core: An Evolving Community-Developed Biodiversity Data Standard. PLoS ONE.

[B3339696] Ytow NOZOMI, Morse D. R., Roberts D. M. (2001). Nomencurator: a nomenclatural history model to handle multiple taxonomic views. Biological Journal of the Linnean Society.

[B3339706] Ytow N, Morse D. R., Roberts D. M. (2006). Rough Set Approximation as Formal Concept. Journal of Advanced Computational Intelligence and Intelligent Informatics.

